# Dose response association of objective physical activity with mental health in a representative national sample of adults: A cross-sectional study

**DOI:** 10.1371/journal.pone.0204682

**Published:** 2018-10-24

**Authors:** Paquito Bernard, Isabelle Doré, Ahmed-Jérôme Romain, Gabriel Hains-Monfette, Celia Kingsbury, Catherine Sabiston

**Affiliations:** 1 Department of Physical Activity Sciences, Université du Québec à Montréal, Montréal, Québec, Canada; 2 Research Center, University Institute of Mental Health at Montreal, Montréal, Québec, Canada; 3 Research Center, Centre Hospitalier de l'Université de Montréal (CRCHUM), Montréal, Québec, Canada; 4 University of Toronto, Faculty of Kinesiology and Physical Education, Toronto, Ontario, Canada; Maastricht University, NETHERLANDS

## Abstract

Although higher physical activity (PA) levels are associated with better mental health, previous findings about the shape of the dose–response relationship between PA and mental health are inconsistent. Furthermore, this association may differ according to sedentary levels. We investigated the cross-sectional dose-response associations between objectively measured PA and mental health in a representative national sample of adults. We also examined whether sedentary time modified the PA—mental health associations. Based on 2007–2013 Canadian Health Measures Survey data, PA and sedentary time were measured using accelerometry among 8150 participants, aged 20 to 79 years. Generalized additive models with a smooth function were fitted to examine associations between minutes per day of moderate and vigorous PA (MVPA), light PA (LPA), daily steps (combined or not with sedentary time) and self-rated mental health. A significant curvilinear relationship between average daily minutes of MVPA and mental health was observed, with increasing benefits up to 50 minutes/day. For LPA, a more complex shape (monotonic and curvilinear) was found. For daily steps, inverted U-shaped curve suggested increasing benefits until a plateau between 5 000 and 16 000 steps. The MVPA-LPA combination was significantly associated with mental health but with a complex pattern (*p* < 0.0005^E-06^). The tested PA-sedentary time combinations showed that increasing sedentary time decreased the positive PA-mental health associations. Non-linear dose-response patterns between the PA modalities and self-reported mental health were observed. Optimal doses of daily minutes of MVPA, LPA, MVPA combined with LPA and daily steps are independently associated with better mental health in adults. The results also suggest that PA-mental health associations could be hampered by daily sedentary time.

## Introduction

The past decade has seen the rapid development of public mental health action plans by the World Health Organization [1–3]. Mental health has been included as one of the Sustainable Development Goals of the United Nations [4]. Broadly defined, mental health is ‘‘a state of well-being in which every individual realises his/her potential, can cope with the normal stresses of life, can work productively and fruitfully, and is able to make a contribution to her or his community” [1]. A generally well-supported principle is that there is no health without mental health [5–7]. Hence, identifying factors that strengthen mental health and reduce the risk of mental illness and symptoms is a public health priority [8].

Physical activity (PA) is a health behavior consistently associated with mental health in adults [9,10]. Specifically, regular PA significantly decreases the risk of developing depression [11], and mental disorders in healthy adults [12] or adults with chronic disease [13] and decreases symptoms in individuals with psychiatric disorders [14]. The protective effects of PA has been demonstrated in cross-sectional [15] and longitudinal [16,17] study designs. In two different population-based representative samples, light [18] and moderate to vigorous [19], PA assessed objectively was associated with lower psychological distress. These findings demonstrate that PA is associated with both reduced symptoms and incidence of mental illness and improved positive mental health.

To inform public health interventions, the dose of PA providing mental health benefits needs to be identified. However, the dose–response relationships between PA and self-reported mental health, and the shape of this association remain unclear and debated [20]. Understanding this dose-response question would help to identify the minimum or maximum of PA that provides mental health benefits. In Stephen’s investigation [21], a quasi-linear dose–response relationship between recreational/household PA and well-being, positive affect and depression was revealed in nationally representative US and Canadian cohorts. A recent investigation suggested that an inverted U-shaped curve reflects the association between PA and mental health, with the best benefit from 2.5 to 7.5 hours of weekly PA [22]. Abu et al [23] demonstrated a linear and positive association between self-reported total PA volume (Metabolic Equivalent of Task/hour per week) and mental health using the Mental Health Inventory in European cohorts. In older adults, a S-shaped dose-response was found, suggesting that participants declaring more than 150 minutes of weekly PA had better self-reported mental health [24]. Hamer et al [25] suggested a linear inverse dose-response pattern between number of PA sessions per week and risk of psychological distress in a large cohort of women. Finally, a positive linear dose–response relationship between leisure PA and emotional well-being was reported in US adult cohort [26].

Inconsistencies in findings about the shape of the dose–response relationship between PA and mental health may be partly due to PA assessment [27]. One major limitation is the systematic use of self-reported PA measures (30). In fact, PA can vary by 20%-60% between objectively and self-reported PA measures [28]. Consequently, due to an over estimation of PA and especially for Moderate and Vigorous Physical Activity (MVPA) [29], inaccurate PA doses could be recommended.

Also, the daily pattern of PA in adult populations includes large-bout(s) of Light Physical Activity (LPA) with short-bout(s) of MVPA [30,31]. As such, the different intensities of PA should be explored. When examining the dose response using objectively-assessed PA, Vallance et al [19] reported a linear inverse dose-response between quartiles of daily MVPA minutes and self-reported depressive symptoms. No linear association has been found between daily time spent in LPA and low psychological distress level (17). However, previous studies fail to examine the association between this MVPA-LPA combination and mental health. Furthermore, step counts can be used as an assessment of general PA and there is some evidence to suggest an inverse linear association between daily steps and self-reported depression level among older adults [32]. Finally, objective assessments of PA enable the assessment of sedentary time, whereby higher sedentary duration has been associated with poorer mental health in adults [16,33–35]. The time spent in sedentary behaviors has also been identified as a potential moderator of PA-mental health association [34,36]. Hence, dose-response associations between PA and self-rated mental health may differ according to sedentary time levels.

Given the importance of understanding the cross-sectional dose-response relationships to inform PA guidelines, the study aims were to examine in adults:

1. the shape of the associations between PA volume across intensity levels, daily steps and mental health
2. whether combinations between PA and sedentary time are associated with mental health,
3. whether sedentary time modifies the PA—mental health associations.

## Materials and methods

### Participants and study background

Data were drawn from the Canadian Health Measures Survey (CHMS) cycle 1 to 3 (2007–2013) conducted by Statistics Canada. The CHMS is a national survey representative of approximately 96% of the Canadian population, aged 6 to 79 years, living in private households at the time of the survey [37]. Data were collected in two stages. First, sociodemographic and general health information were collected during an interview at the participants' home. Then, weight and height measurements were collected during a subsequent visit to a mobile clinic. Residents of Indian Reserves, Crown lands, institutions and certain remote regions, and full-time members of the Canadian Forces were excluded [37]. This survey was approved by the Health Canada’s Research Ethics Board [38]. All respondents provided written informed consent. The present study included participants aged 18 to 79 years with complete data for mental health and physical activity. Pregnant women and participants with functional limitation were not included in analyses. More details about method and measures have been previously published [37–39].

### Measures of physical activity and sedentary time

Upon completion of a mobile examination centre visit, participants were asked to wear an Actical accelerometer (Phillips-Respironics, 17 grams, omnidirectional accelerometer) over their right hip on an elasticized belt during their waking hours for 7 consecutive days. The accelerometers started to collect data the day after the mobile examination centre appointment. The monitors were then posted to Statistics Canada. The data were validated by research assistants to determine if they were still within the manufacturer’s calibration specifications. The Actical measures and records time-stamped acceleration in all directions, thereby indicating the intensity of physical activity. The digitized values are summed over an interval of one minute. Accelerometer data were in a count value per minute (cpm) and also into steps accumulated per minute [40]. The Actical is a valid and reliable instrument to measure PA in adults [40]. All data are blind to respondents while they are wearing the device.

Consistent with common analytical procedures for accelerometry, a valid day was defined as 10 or more hours of wear time and respondents with 4 or more valid days were retained for analyses [39]. Accelerometer data were not included in the analyses if a participant had extreme counts (i.e., >20 000 cpm) [41]. The number of minutes per day spent in PA of different intensity levels was categorized using standard cpm for adults: moderate and vigorous (⩾ 1535 cpm), light (100 to 1534 cpm) and sedentary behavior (<100 cpm) [42]. The following variables were separately used in analyses: average minutes per day of MVPA and LPA, average steps per day, and average minutes per day of sedentary behavior.

### Mental health

Self-reported mental health was measured using the following item: “In general, would you say your mental health is: Excellent (coded as “5”), Very Good, Good, Fair, Poor (coded as “1”)?”, which is a widely used item in national population health surveys in eleven countries, including Canada [43]. A previous study using data from the Canadian Community Health Survey suggested that high self-reported meatl health score was significantly associated with a lower health service utilisation and risk of mental health disorder [44]. Concurrent validity has been supported with various mental health indicators, including positive mental health scales (e.g., Well-being index) and depression (e.g., Geriatric Depression Scale) [45].

### Covariates

Sociodemographic covariates included age, sex, daily smoking (yes/no), household income (ranged from ≤15 000 to 100 000$) and education levels (ranged from ≤high school to university). Accelerometer wear time (daily minutes) and season of accelerometer assessment were also computed and included in analyses. Body mass index was computed by measuring an individual’s weight and height.

### Statistical analysis

Generalized additive models (GAMs) were used to examine the shape of the association between mental health, PA and sedentary time. The GAM is an extension of the generalized linear model in that one or more predictors may be specified using a smooth function [46]. GAM is a nonparametric model that allows nonlinear relationships to be modeled with flexibility without specifying the nonlinear functional form. The GAM is estimated using a penalized maximum likelihood procedure—usually iteratively reweighted least squares. Predictions from GAM were plotted with 95% confidence intervals. GAMs can accommodate the combination of two predictors, in a way that is conceptually comparable to interactions in generalized linear models. The joint smooth function was specified for the following combinations: MVPA-LPA, MVPA-sedentary, LPA-sedentary and steps-sedentary. The results are presented in terms of 3-dimensional plots characterizing the associations between mental health and tested combinations. Covariates for all regression analyses included age, sex, education, season, income, body mass index and current smoking status. These covariables are commonly related to PA and sedentary behaviors in adults [47–49]. The wear time (i.e., 24 hours minus nonweartime [> 60 minutes with 0 counts]) can also influence PA and sedentary variables [50], consequently they were included in models. To account for the complex, multistage probability sampling design, the weights provided by the CHMS were used in the analyses (i.e., activity monitor subsample weights combining cycle 1, 2 and 3). All analyses were performed using *survey* [51] and *mgcv* [46] packages in R version 3.3.

## Results

### Sample characteristics

Accelerometer data from the three cycles were available from 8150 participants (rounded to the nearest 10 as per Statistics Canada confidentiality requirements) and were included in the current study, representing about 24 942 139 Canadian residents. The mean of self-reported mental health was 4.0 (Standard error [SE] = 0.1, Median [Mdn] = 3.9). The mean age was 44 years (SE = 0.4) and 50.3% were women. On average, participants spent 21.3 (SE = 0.4, Mdn = 15.4), 214.3 (SE = 1.5, Mdn = 204.6) and 508 (SE = 3.3, Mdn = 561.3) minutes per day of MVPA, LPA and sedentary time, respectively. Data from twelve outliers were excluded because of extreme accelerometer data. Table 1 shows weighted characteristics of participants included in the analyses. More descriptive results about PA are available in Supplementary file (S1 Table).

**Table 1:** Weighted means and ratio among participants.

	Total
Age (years)–Mean (M) (SE)	44.7 (0.4)
Men	44.9 (0.4)
Women	44.6 (0.4)
Sex % (N)	
Men	49.7 (12 396 243)
Women	50.3 (12 545 896)
Current daily smoker % (N)	20.5 (5 113 138)
Education % (N)	
Less than high school diploma	7.1 (1 770 892)
High school diploma	9.9 (2 469 272)
Trade certificate or diploma	16.7 (4 165 337)
College, non-univ diploma	8.9 (2 219 850)
University diploma	13.2 (3 292 362)
Bachelor's degree	12.8 (3 192 594)
Univ certificate/diploma	2.3 (573 669)
Not Stated	28.8 (7 183 336)
Household incomes (Canadian dollars) % (N)	
<15 000	7.5 (1 870 660)
15 000 < 30 000	17.2 (4 290 048)
30 000 < 45 000	18.1 (4 514 527)
45 000 < 80 000	27.2 (6 784 262)
> 100 000	30 (7 482 642)
Marital status % (N)	
Living with someone, married	66.1 (16 486 754)
Separated, widowed, divorced	33.9 (8 455 345)
Occupation % (N)	
Yes	79.2 (19 754 174)
No	20.8 (5 187 965)
Self-reported health—M (SE)	2.6 (0.1)
Self reported chronic disease % (N)	25.2 (6 285 419)
Body mass index (kg/m^2^)—M (SE)	26 (0.1)
MVPA (min/d)—M (SE)	21 (0.4)
LPA (min/d)—M (SE)	214 (1.5)
Sedentary (min/d)—M (SE)	508 (2.3)
Steps (steps/d)—M (SE)	7817 (69.5)
Wear time—(min/d)—M (SE) (valid days only)	784 (2.4)
Season of assessment % (N)	
Autumn	32 (7 981 484)
Spring	25 (6 235 535)
Summer	22 (5 487 271)
Winter	21 (5 237 849)

### Shape of associations between mental health and physical activity

**Fig 1:**
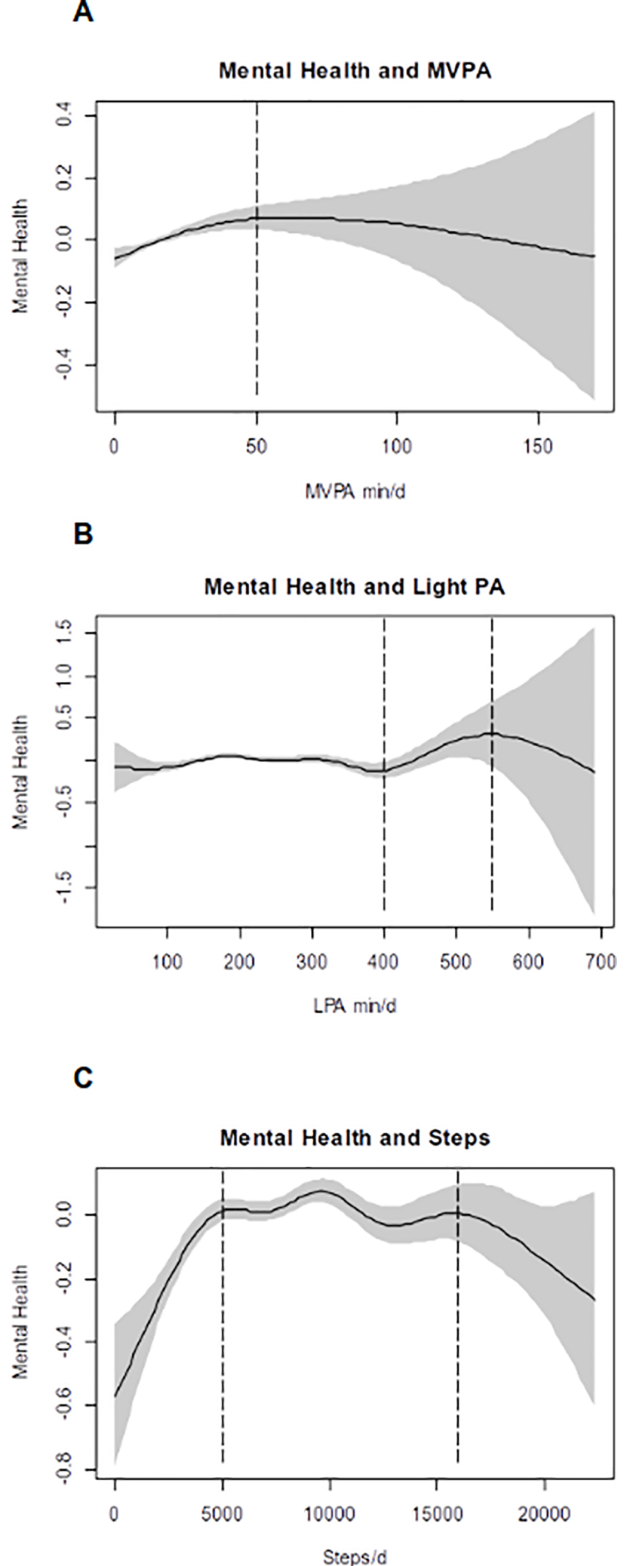
Associations between self-reported mental health and moderate-vigorous physical activity, light physical activity and daily steps. Notes. MVPA = Moderate and Vigorous Physical Activity, LPA = Light Physical Activity. Each model is weighted and adjusted for age, sex, education, wear time, season, income, body mass index, and current smoking status.

Fig 1 presents GAM results from adjusted models of mental health as a function of each daily MVPA, LPA duration, and number of steps. The lines show the smoothed function from GAM for PA variables, and the shaded area indicates the 95% confidence interval. Each model is adjusted for age, sex, education, wear time, season, income, and current smoking status. The three PA variables were significantly associated with self-rated mental health. A visual examination of the plots indicated nonlinear associations between all three PA modalities and self-reported mental heath. A significant curvilinear relationship between average daily minutes of MVPA and mental health (*p <* 0.0001, Adjusted R^2^ = 0.05) with upper limit (≈ 50 minutes) was found (Fig 1A). Higher MVPA dose (> 50 minutes) was associated with lower mental health level. For LPA, a significant association with self-reported mental health (*p <* 0.001, Adjusted R^2^ = 0.05) was observed with a more complex shape. The Fig 1B indicated a general monotonic form from 0 to 400 minutes of daily LPA, and above 400 minutes, there was a curvilinear form observed. A visual examination of the plot indicated that there was a better mental health for participants engaged in around 400 to 550 average minutes of daily LPA.

**Fig 2:**
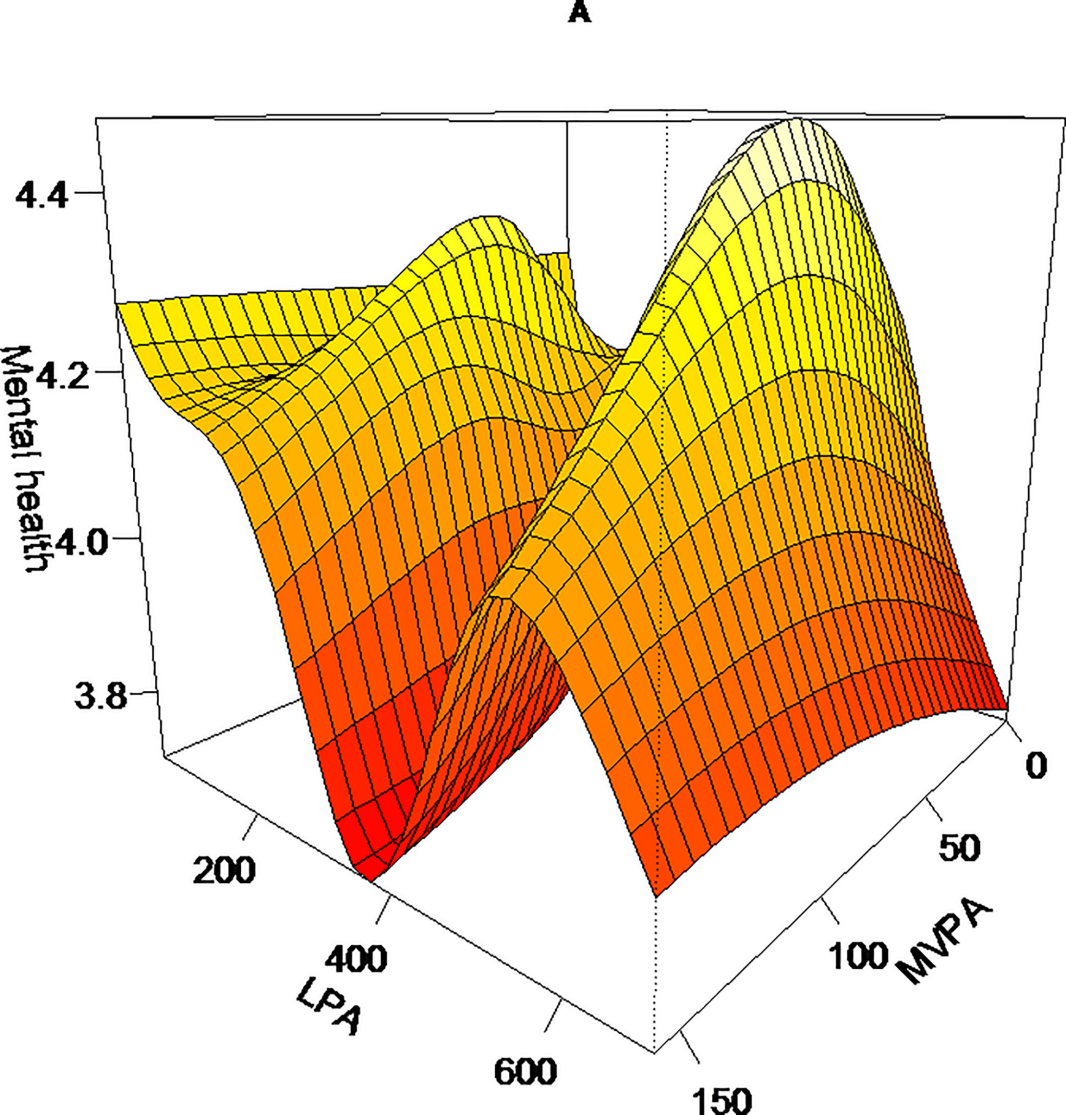
Graphic representation of associations between self-reported mental health and MVPA-LPA combination. Notes: Higher height of the surface indicates better level of a mental health. Fig 2 *p* = 0.0005E-05, Adjusted R^2^ = 0.06. MVPA = Moderate and Vigorous Physical Activity, LPA = Light Physical Activity. The model is weighted and adjusted for age, sex, education, wear time, season, income, body mass index, and current smoking status.

The significant association between daily steps and mental health (*p <* 0.0001, adjusted R^2^ = 0.06) was plotted as an inverted U-shaped curve suggesting an increase of benefits until a plateau between 5 000 and 16 000 steps (*p* < 0.0001, adjusted R^2^ = 0.06) (see Fig 1C). Highest mental health levels were observed between 5 000 and 16 000 average daily steps. MVPA-LPA combination was also significantly related with mental health (*p <* 0.0001, adjusted R^2^ = 0.06). Specifically, the associations between combinations of PA modalities with self-reported mental health are presented in Fig 2. Fig 2 shows a complex relationship between MVPA and LPA in association with mental health. Indeed, lower doses of LPA combined with higher doses MVPA, or inversely, were associated with better mental health (i.e., assessed as positive for participants). The combinations of high MVPA-low LPA doses, and low MVPA-high LPA doses, were associated with better mental health. However, LPA daily dose ranging from 200 to 350 minutes was associated with a low mental health level, regardless of MVPA dose. The extreme MVPA-LPA combined doses were associated with more moderate level of mental health scores.

### Shape of associations between mental health and physical activity-sedentary time combination

**Fig 3:**
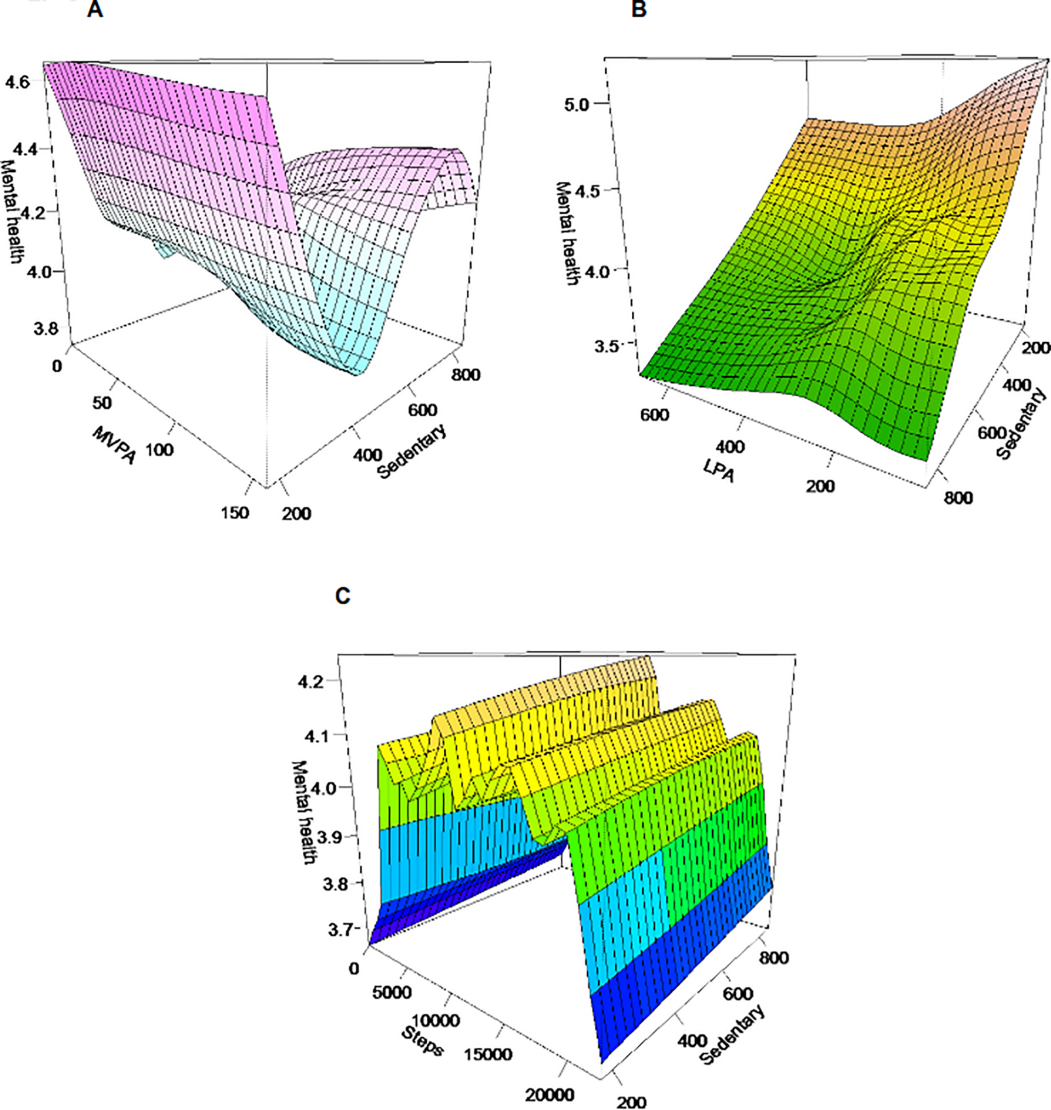
Graphic representations of associations between self-reported mental health and tested combinations of physical activity modalities. Notes: Higher height of the surface indicates better level of a mental health. (A) *p* = 0.0009E-03, Adjusted R^2^ = 0.06. (B) *p* = 0.0002E-01, Adjusted R^2^ = 0.06. (C) *p* = 0.0002E-08, Adjusted R^2^ = 0.06. MVPA = Moderate and Vigorous Physical Activity, LPA = Light Physical Activity.Each model is weighted and adjusted for age, sex, education, wear time, season, income, body mass index, and current smoking status.

The three PA modalities (i.e., MVPA, LPA, steps) combined with sedentary time were significantly associated with mental health. Fig 3A, 3B and 3C allow us to see how non-linear associations observed in Fig 1 were modified by average daily sedentary time. The plots show each individual PA variable on the x-axis, sedentary time on the y-axis and mental health on the vertical z-axis. Higher height of the surface indicates better level of a mental health (i.e., assessed as (very) good, or excellent). Regarding MVPA (Fig 3A), when sedentary time increased, a higher MVPA dose was necessary to obtain similar mental health level. Moreover, when sedentary time increased, benefits associated with MVPA on mental health persisted but indicate various magnitudes of mental health level. However, MVPA was associated with better mental health at any dose for lower time spent in sedentary behaviors.

For the LPA-sedentary combination (Fig 3B), when sedentary time decreased, LPA was related to higher self-reported mental health. For lower sedentary time, low dose of LPA was associated with high level of mental health. Regarding the number of daily steps-sedentary time combination, the inverted U-shaped association between daily steps and mental health level remained but became somewhat more pronounced while sedentary time increased (see Fig 3C). Four different perspectives of each graph in Fig 3 are available in Supplementary file (S1–S4 Figs). The estimated regression coefficients for all models are reported in Supplementary file (S2 Table).

## Discussion

### Main findings and comparison with previous literature

The present study demonstrated a non-linear dose-response pattern between the PA modalities (i.e., MVPA, LPA, MVPA-LPA combination and steps) and self-reported mental health. This study expanded on previous research by using objective PA measure to assess dose-response association with mental health. CHMS’s participants had similar sociodemographic characteristics and time spent in daily PA and sedentary activities compared to adults from US National Health and Nutrition Examination Survey (NHANES) [19]. Increasing mental health levels were observed from the first MVPA minute until 50 minutes. The identified MVPA shape is consistent with findings obtained with self-reported PA data in a previous population study [52]. However, this finding differs from Vallance investigation’s [19] using objective data in NHANES.

Our findings related to LPA suggested that mental health levels were significantly higher after 400 minutes (≈6h30). The shape between LPA and mental health is consistent with evidence of no linear association between objectively assessed LPA and psychological distress [18]. Surprisingly, a zero association is noted for lower LPA duration. This finding may be explained, at least in part, since the LPA is both leisure and occupational activity and there is some proposition that the health benefits from leisure LPA can be hampered by occupational LPA [53]. Future research efforts are needed to better understand the health outcomes associated with different forms of LPA.

Benefits for mental health increased between 1 and 5 000 steps, with a plateau until 16 000 steps. Our findings differ from Ewald’s [32] that found a lower depression score with increased steps per day but with an inflection point at 8 000 steps. However, this investigation included urban-dwelling adults.

Findings combining PA variables and sedentary time suggest that, when the two behaviors coexist, more MPVA time in sedentary context is required to maintain a high mental health level. This complex significant association supports previous evidence showing that the associations between MVPA and psychological distress [35] or well-being differ according to sitting time [16,34]. A ‘hollow’ V-shaped curved was noticeable in Fig 2B. This rapid decrease of mental health could be artificial or confounded by job type [34]. This ‘hollow’ is equivalent to ≈450 minutes (approximately 7.5 hours); i.e., a workday for an office worker. The occupational type is a major determinant of objective sedentary time [54,55].

A visual examination of Fig 2C, suggests that LPA—mental health association is mainly related to sedentary time. The monotonic and curvilinear shape of LPA–mental health association was different in the context of lower sedentary behavior. Indeed, the highest level of positive mental health was related to the lowest dose of sedentary time and LPA. In other words, an inverse association between LPA and mental health could be found in the context of very low time spent in sedentary activities. This result is inconsistent with previous investigations showing that the sedentary and psychological distress association was apparent for longer time spent in sedentary activities [18,56].

### Interpretation of main findings

These findings have important implications for public health, by highlighting that other PA modalities other than MVPA are important for psychological benefits. For instance, walking or self-paced running are well-accepted activities in general population [57] and clinical populations (18) and their effects on mental health were apparent as early as 5 000 steps per day. Consequently, finding from the present study provide support for PA as a mental health promotion tool that is valuable at light, moderate, vigorous intensities (combined or not). It is also important to advocate for immediate possible benefits from first steps of walking/running rather than specific dose to achieve because inactive adults are more likely to engage in lower duration of PA [58–61].

LPA and MVPA were differentially associated with self-reported mental health. This finding supports indirect evidence that the complex PA-mental health association could not only be driven by physiological processes. Indeed, social, psychological and genetics factors have been identified as potential mediators [62], and parallel processes could be involved depending on PA modalities.

The results also suggest that the association between the LPA-MVPA combination and mental health could be hampered by daily sedentary time. The LPA-mental health association was the most variable in sedentary context. Thus, the “move more and sit less” public health strategy [61,63], arguing in favor of interventions targeting PA and sedentary behaviors could be also favorable for mental health. In research perspective, the PA sedentary time interactions with mental health outcomes should be more systematically investigated.

### Strengths and limitations

This is the first time that data from representative national sample of adults has been used to explore the dose-response relationship between self-reported mental health and objectively measured PA. Thus, our findings are generalizable to the general Canadian population from early adulthood to older adults. It is also the first investigation to examine the contribution of sedentary time on PA-mental health association. Additionally, this study used a non-linear statistical modelling approach, which allowed the data to fully inform the shape of the PA-mental health associations. The major limitation of this study is the cross-sectional design that precludes inferences about cause-effect relationships. Indeed, PA could lead to better mental health, higher mental health level could induce one to engage in PA, or mental health and PA could also be to some third variable (e.g., social status). Furthermore, the explained variance in all adjusted models was low (<10%). Although, mental health was measured with a valid item [64], a comprehensive assessment of mental health including various dimensions of well-being might influence the dose-response relationships observed in this study. Although accelerometers have the benefit of measuring intensity of PA, no information were available on the PA domains or contexts; hence, we are unable to determine whether the association with these PA modalities varies according to social context or underlying motivation for PA [65,66]. It is important because PA domains has been shown to influence the magnitude of PA volume and mental health association [67]. Finally, daily PA means were used as the main independent variables because participants wore an accelerometer between 4 and 7 days. Consequently, the identified PA dose may be different because we could not control for the weekly PA accumulation. In other words, it may be inappropriate to design recommendations by multiplying daily PA doses.

## Conclusion

These empirical findings make a unique contribution in the literature by providing a new understanding of PA-sedentary time interplay with mental health, using objective measures and population-level data. From a public mental health perspective, four messages can be drawn from this study: every daily MVPA minutes until 50 minutes are positively associated with mental health; every daily steps are positively associated with mental health and more than 5 000 are suggested for increased benefits; when LPA is combined with MVPA, greater benefits for mental health might be achieved and the “move more and sit less” strategy might also applied to mental health. Future prospective general population studies using objective measures of PA and sedentary time should investigate the protective effect of different PA doses on mental health.

## Supporting information

S1 Fig Associations between self-reported mental health and MVPA-LPA combination. (PDF)

S2 Fig Associations between self-reported mental health and MVPA-Sedentary combination. (PDF)

S3 Fig Associations between self-reported mental health and LPA-Sedentary combination. (PDF)

S4 Fig Associations between self-reported mental health and Steps-Sedentary combination. (PDF)

S1 Table Descriptive data about physical activity and sedentary. (PDF)

S2 Table Weighted estimated regression coefficients for physical activity and mental health associations for each model. (PDF)
