# Supplementary material for: Dose response association of objective physical activity with mental health in a representative national sample of adults: A cross-sectional study
Source: PLoS One. 2018 Oct 24;13(10):e0204682. doi: 10.1371/journal.pone.0204682 (PMC6200189; doi:10.1371/journal.pone.0204682)

S1 Fig. Associations between self-reported mental health and MVPA-LPA combination (It is the same 3D graphic presented with four different perspectives)

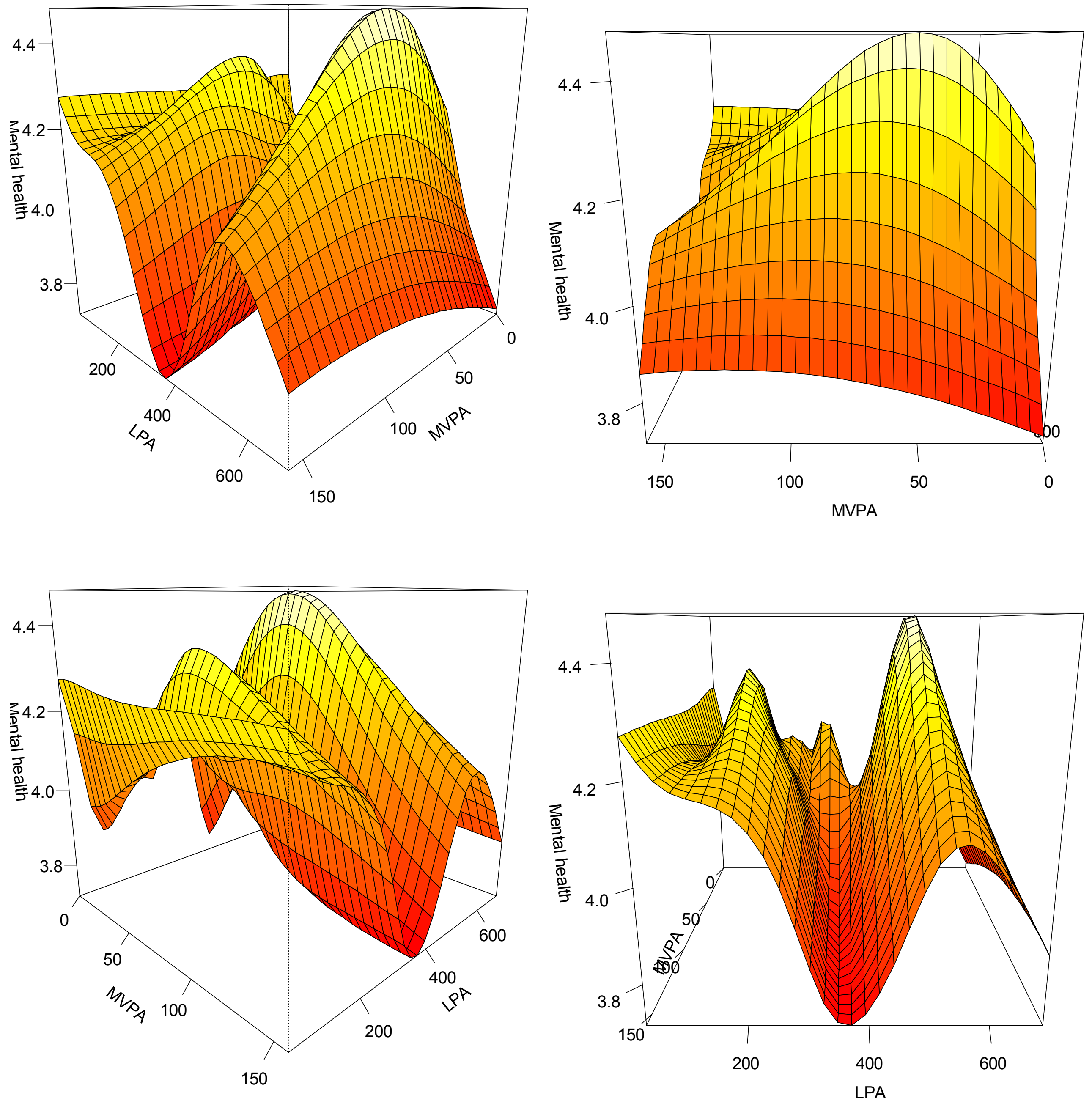

Supplement: S1 Fig — (PDF) [file pone.0204682.s001.pdf]
