# Supplementary material for: Dose response association of objective physical activity with mental health in a representative national sample of adults: A cross-sectional study
Source: PLoS One. 2018 Oct 24;13(10):e0204682. doi: 10.1371/journal.pone.0204682 (PMC6200189; doi:10.1371/journal.pone.0204682)
Supplement: S1 Table — (PDF) [file pone.0204682.s005.pdf]

S1 Table. Descriptive data about physical activity and sedentary

	MVPA (min/d)		LPA (min/d)		Steps		Sedentary (min/d)	
	M (SE)	Mdn (IQR)	M (SE)	Mdn (IQR)	M (SE)	Mdn (IQR)	M (SE)	Mdn (IQR)
Sex								
Men	23.8 (0.6)	18.4 (26.7)	219.6 (2.4)	208.1 (113.4)	8334 (109)	7842 (4965)	538.9 (3)	545.1 (129.3)
Women	18.7 (0.5)	13.6 (21.6)	209.8 (1.9)	201.6 (95.4)	7365 (88)	6910 (4377)	557.4 (2.8)	561.8 (123.9)
Smoker								
Yes	18.5 (0.5)	11.7 (24)	218.3 (4.3)	201 (121.1)	7490 (76)	6895 (4845)	539.5 (5)	542.5 (146.9)
No	21.9 (0.8)	16.4 (22.2)	213.8 (1.6)	205.4 (100)	7942 (180)	7540 (4446)	550.2 (2.3)	557.6 (123.7)
Marital status								
Living with someone, married	19.6 (0.5)	14.3 (21.3)	221.7 (1.9)	211.7 (103.2)	7893 (84.9)	7452 (4527)	553.1 (2.4)	559.4 (121.6)
Separated, widowed, divorced	24.4 (0.8)	18.4 (30.1)	201.1 (2.5)	188.9 (98.1)	7767 (127)	7252 (4425)	538.4 (3.9)	542.2 (135.5)
Occupation								
Yes	23.3 (0.5)	18.1 (24.3)	223 (1.9)	211.4 (102.5)	8254 (83.1)	7827 (4409.4)	540.7 (2.5)	547.4 (129.5)
No	14.6 (0.7)	7.9 (18.3)	189.4 (2.6)	184.1 (100)	6514 (126.8)	5978 (4275.2)	572.8 (3.5)	570.7 (114.4)
Chronic disease								
Yes	25.7 (0.9)	20.4 (25.6)	226.1 (2.7)	215 (441.4)	8557 (130.2)	8117 (4446.1)	552 (2.5)	556.7 (125)
No	18.5 (0.4)	13.4 (21.7)	207.2 (1.9)	197 (104.6)	7435 (82.7)	7028 (4621.6)	541.6 (3.6)	549.9 (129.1)
