# Supplementary material for: Dose response association of objective physical activity with mental health in a representative national sample of adults: A cross-sectional study
Source: PLoS One. 2018 Oct 24;13(10):e0204682. doi: 10.1371/journal.pone.0204682 (PMC6200189; doi:10.1371/journal.pone.0204682)
Supplement: S2 Table — (PDF) [file pone.0204682.s006.pdf]

S2 Table. Weighted estimated regression coefficients for physical activity and mental health associations for each model

	Estimate	*Df*	*F*	*p*	*Adjusted R^2*
MVPA	2.2	2.8	6.0	0.0007	0.05
LPA	7.2	8.2	3.0	0.003	0.05
Steps	6.8	7.9	7.8	0.0003E-06	0.06
MVPA*LPA	24.4	27.7	3.6	0.0005E-06	0.06
MVPA*sedentary	18.8	23.9	2.6	0.0009E-03	0.06
LPA*sedentary	21.6	26.4	2.5	0.0002E-01	0.05
Steps*sedentary	18.6	23.4	3.7	0.0002E-08	0.06
